# Bizarre Parosteal Osteochondromatous Proliferation of the Hand: A Report of an Atypical Case and Current Concepts

**DOI:** 10.7759/cureus.56392

**Published:** 2024-03-18

**Authors:** Grigorios Kastanis, Anna Pantouvaki, Mikela-Rafaella Siligardou, Ioannis M Stavrakakis, Petros Kapsetakis

**Affiliations:** 1 Orthopaedics, Venizelio General Hospital of Heraklion, Heraklion, GRC; 2 Physiotherapy, Venizelio General Hospital of Heraklion, Heraklion, GRC

**Keywords:** bpop, benign hand tumor, phalangeal tumor, bizarre osteochondromatous proliferation, nora’s lesion

## Abstract

Bizarre parosteal osteochondromatous proliferation (BPOP), or Nora’s lesion, is an unusual, benign, bony lesion often found in the tubular small bones of the hand and foot. In general, two characteristic radiological signs are used to diagnose the lesion, namely, (1) the absence of corticomedullar continuity and (2) BPOP developed from the parosteal surface of bones with an intact underlying cortex. Here, we present an atypical case of Nora’s lesion of the proximal phalanx of the index finger, in which BPOP was diagnosed only histologically, with preoperative imaging examinations (X-ray and MRI) suggesting another lesion (enchondroma). Therefore, imaging (X-ray and MRI) alone may be inadequate to achieve the correct diagnosis of the lesion because many cartilaginous neoplasms may surround a broad range of lesions that mimic BPOP. Only histopathological evidence can confirm the correct diagnosis of the lesion.

## Introduction

Bizarre parosteal osteochondromatous proliferation (BPOP), a rare, benign, exostotic bony lesion, affects the surfaces of the small tubular bones of the hand and foot [1]. Nora et al. (1983) first described this proliferative lesion with radiographic findings showing a heavy calcific mass with a broad base originating from the underlying cortex of the small tubular bones of the hand and foot, but an intact cortex of the involved bone [2].

To date, 322 BPOP cases with a histopathological diagnosis have been reported in the literature, in which this benign lesion did not originate exclusively from the small tubular bones of the hand and foot but was also observed in other parts of the human skeleton [1]. In a systematic review, Ipponi et al. (2022) described that tubular bones of the hand are most frequently affected constituting 191 (59.3%) cases. They reported the following distribution: metacarpal bones (38 cases); proximal (41 cases), middle (44 cases), and distal phalanges (19 cases); and unknown exact location (49 cases). Other bones of the upper limb demonstrate a lower occurrence, including the radius (10 cases), ulna (17 cases), humerus (four cases), and clavicle (one case) [1]. The second most common location for BPOP was observed in the bones of the foot (66 cases), while other sites of the human skeleton that have been described in various case reports or case series were the spine (one case), femur (11 cases), patella (one case), tibia (eight cases), fibula (four cases), mandible (four cases), maxilla (one case), zygoma (one case), nose (one case), and an unspecified location in the skull (one case) [1,3,4].

Patients at the time of diagnosis tend to be between the third and fourth decade of life, with a mean age of 34.3 years, although diagnosis at younger ages is not infrequent [3]. An equal distribution of the disease by sex has been observed (49.8% females vs. 50.2% males) [4]. Diagnosis of BPOP is based on clinical presentation and symptoms (pain, swelling, or functional restriction of motion), or may be incidentally observed during radiographic examination for other diagnostic objectives. Although the final diagnosis of BPOP is classically made through histopathological examination, X-ray imaging has a central role in diagnosis [1]. Barrera-Ochoa et al. (2012) reported that cartilaginous neoplasms enclose diverse lesions with differing clinicopathologic behavior and suggested that Nora’s lesions are easily misdiagnosed [5]. Three reasons for the misdiagnosis of Nora’s lesion have been described, namely, high rate of recurrence (20-55%) as they can be considered as another type of malignant tumor, potential for rapid growth, and atypical histological appearance [3,5].

## Case presentation

**Figure 1:**
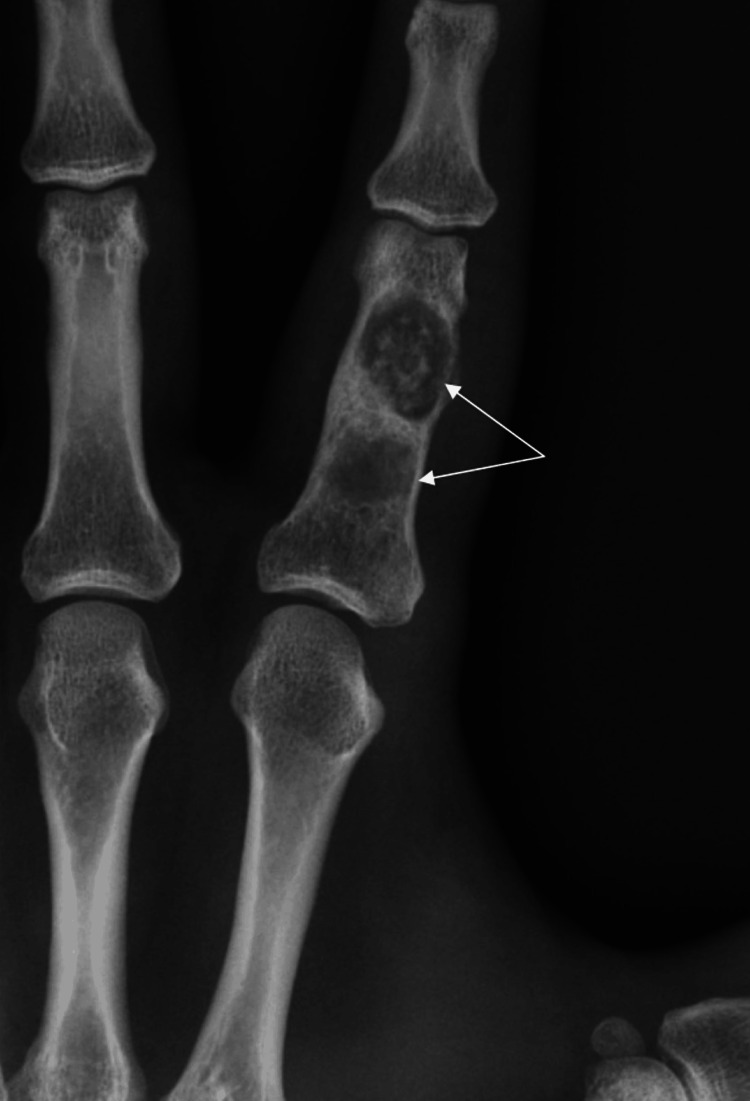
Preoperative X-rays (anteroposterior view) indicating the intraosseous lesions in the proximal phalanx of the index finger of the left hand (white arrows).

**Figure 2:**
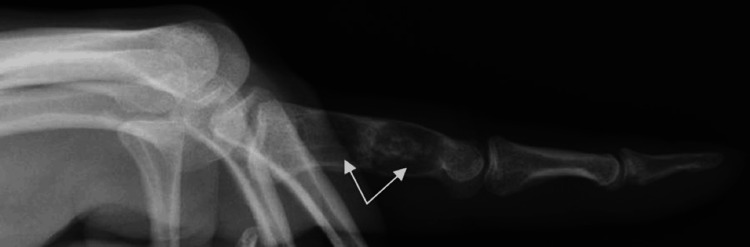
Preoperative X-rays (profile view) indicating the intraosseous lesions in the proximal phalanx of the index finger of the left hand (white arrows).

**Figure 3:**
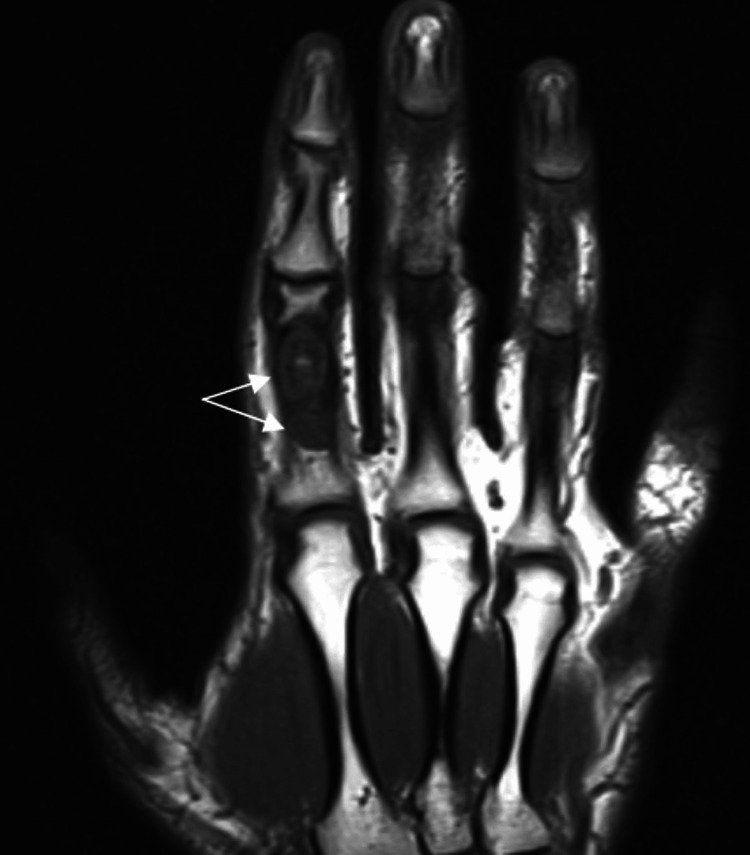
Preoperative MRI (T1 coronal) indicating double intraosseous lesions in the proximal phalanx of the index finger (white arrows).

**Figure 4:**
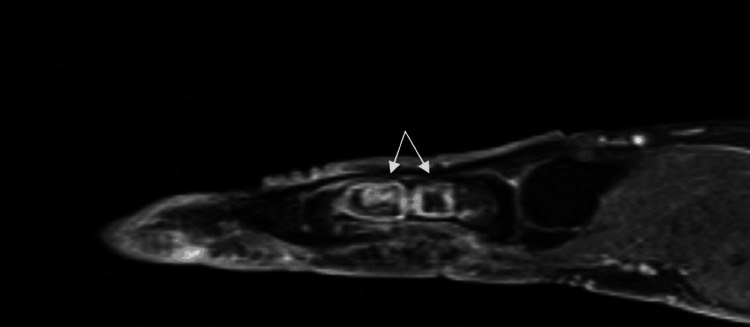
Preoperative MRI (T1 sagittal) indicating double intraosseous lesions in the proximal phalanx of the index finger (white arrows).

A 40-year-old manual worker was admitted to our emergency department with pain and impaired range of motion of the index finger of the left hand. The patient, 24 hours prior, while attempting to tie his shoelaces, had heard a crack sound and felt pain in the proximal phalanx of his index finger. The dominant hand was affected. Clinical examination revealed a painful metacarpophalangeal and proximal interphalangeal joint during range of motion, localized pain, and mild swelling on the dorso-radial side of the proximal phalanx of the index finger. An initial radiographic examination (anteroposterior and profile views) of the affected left hand revealed two separate well-circumscribed metaphyseal lytic lesions in the medullary canal of the proximal phalanx (Figures 1, 2). The distal lesion extended toward the cortex, which showed thinning and pathological fracture, as well as on the radial side of the bone. According to the radiological appearance of the lesion, our initial suspected diagnosis was enchondroma. We suggested that the patient undergo an MRI to validate the diagnosis and aid in determining a preoperative plan for the surgical procedure. The MRI described a well-circumscribed lobulated intramedullary lesion of the proximal phalanx with low signal in T1 and the presence of central calcification. In fat-suppressed sequences, the lesion appeared with a high signal, which confirmed the initial radiological suspicion. Therefore, after informing the patient, we proceeded with surgical curettage of the benign lesion (Figures 3, 4).

**Figure 5:**
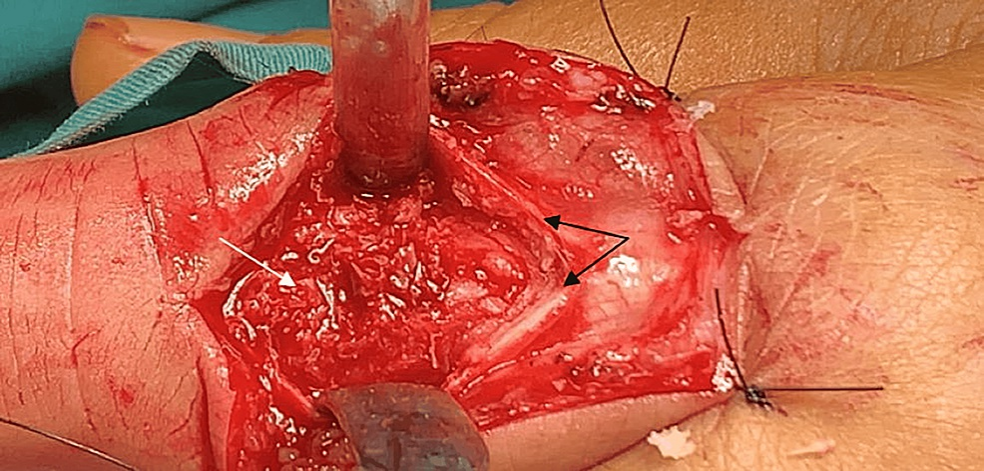
Dorsal aspect of the proximal phalanx with autograft. Two ends of the extensor tendon (black arrows) after the split and dorsal cortex of the proximal phalanx with autograft (white arrow).

**Figure 6:**
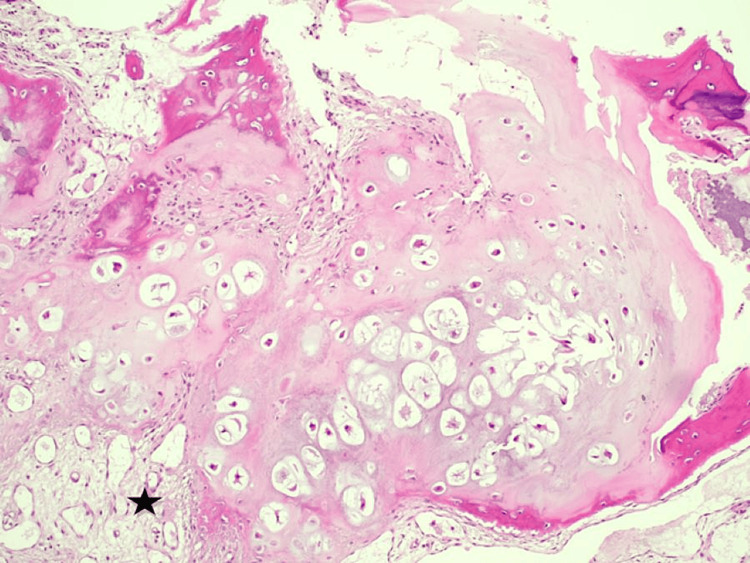
Atypical (bizarre) chondrocytes with enlarged nuclei. Marked hyperchromasia or atypical mitosis is not seen. Hypervascular tissue with spindle cell proliferation, without atypia, between bony trabeculae (star) (hematoxylin and eosin, ×100).

With the wide-awake local anesthesia and no tourniquet technique, we made a 5 cm straight incision over the dorsal aspect on the proximal phalanx of the index finger. While dissecting the subcutaneous tissue, we recognized the extensor tendon apparatus and split the tendon along its midline longitudinal fibers. Initially, we determined the limits of the lytic lesions. The cortex of the bone was extremely thin; notably, the distal cyst had created a fracture on the dorsal surface of the cortex. A wide excision was performed, and the residual intraosseous bone cavity was covered with an autograft from the corresponding distal radius (Figure 5). After wound closure, we applied a palmar cast for three weeks to protect the phalanx because we believed that no osteosynthesis was required. The histopathological diagnosis was BPOP. On hematoxylin and eosin staining, the cartilage appeared to have irregular maturation in the bone that produced chondro-osteoid with characteristic basophilic tinctorial quality (blue bone) and atypical (bizarre) chondrocytes with enlarged nuclei. Marked hyperchromasia or atypical mitosis was not seen. Hypervascular tissue with spindle cell proliferation, without atypia, between bony and trabeculae was also described (Figure 6).

After three weeks, passive exercises and manual therapy were initially performed to increase soft tissue flexibility and range of motion. At the final follow-up at six months, the patient had a painless full range of motion without any restriction or recurrence.

## Discussion

BPOP, or Nora’s lesion, is a rare, benign reactive lesion arising from the periosteum through cartilaginous metaplasia [3]. This lesion commonly affects small tubular bones, such as the proximal and middle phalanxes, and metacarpal and metatarsal bones; the hand is affected four times more frequently than the foot [2,3]. Because of its osteochondromatous origin, BPOP diagnosis is challenging, given its rare presentation and radiological and clinical similarity to other osteochondromatous tumors. Protocols approach BPOP diagnosis in two steps: first, the characteristic morphology of the lesion is investigated through X-ray imaging; second, biopsy and histological examination are mandatory for the definitive diagnosis of the lesion [1].

Radiographically, Nora’s lesion is observed as a pedunculated or broad-based osseous protuberance along the cortical surface of the bone, which in most cases involves metaphysis of the involved bone. The underlying bone cortex retains a normal appearance without evidence of medullary invasion, reactive sclerosis, or periostitis [6]. Cortical erosion has occasionally been reported [1]. Other disorders with similar characteristics to this typical appearance include osteochondroma, myositis ossificans, turret exostosis, parosteal osteosarcoma, periosteal chondroma, parosteal chondrosarcoma, and florid reactive periostitis ossificans [1,6].

Barrera-Ochoa et al. (2012) have indicated that X-ray imaging alone cannot determine the presence or absence of medullary continuity of the lesion, which can be established only from axial CT scanning or MRI [5]. Rybak et al. (2007) have presented four cases of BPOP in which corticomedullar continuity with the underlying bone was demonstrated on imaging. The authors indicated that the radiological findings once considered critical for distinguishing BPOP cannot reliably identify or exclude this lesion [6].

Axial CT scanning can validate the calcific nature of the mass and exclude signs of continuity with the medullary canal or interruptions of the cortex. In addition, this method provides a picture of the neighboring soft tissues and can indicate the absence of parosteal irritative reactions [1,5]. On MRI, Nora’s lesion appears as an extramedullary homogeneous region with low signal intensity on T1- T1-weighted sequences, whereas on T2-weighted and short tau inversion recovery sequences, the lesions exhibit slightly increased central signal intensity, with their periphery being of higher signal intensity [1]. In our case, MRI described a well-circumscribed lobulated intramedullary lesion of the proximal phalanx with low signal in T1 accompanied by central calcification and high signal intensity on fat-suppressed sequences. The MRI findings in our case deviated from those typically associated with BPOP, as described in the literature. Therefore, histopathological examination was crucial for achieving an accurate diagnosis.

Histopathology of BPOP involves three components, namely, cartilage, bone, and fibrous tissue, which are present in varying quantities [1]. The cartilage usually forms a cap; less often, it is arranged in lobules separated by dense fibrous tissue with irregular maturation into bone [1,7]. The bone has a characteristic dark blue pigmentary quality, particularly at the interface with the cartilage, whereas the intertrabecular spaces contain proliferating spindle cells that lack cytological atypia [7]. The cartilaginous component is hypercellular and comprises irregular groups of binucleated and “bizarre” chondrocytes [1,7]. Although double-nucleated chondrocytes are common, hyperchromasia and cytological atypia do not develop [1,7].

Although the pathophysiology of Nora’s lesion is unknown, many researchers have suggested that its presence is associated with trauma within two months to three years before its appearance in 30% of cases [3,6]. Post-traumatic calcification of the soft tissues has been described in the literature, and the periosteum can theoretically calcify as a result of a reparative process that follows deep hematomas or localized flogistic events [1].

However, other researchers consider this possibility controversial. Nora et al. (1983), in a case series, have reported that not all cases had a history of trauma, and Ipponi et al. (2022), in a review of 317 cases, have reported that 14.7% had a history of trauma, whereas the remaining 85.3% did not report any significant traumatic events [1].

Other hypotheses regarding the etiology that do not support the trauma theory include that BPOP might be an intermediate stage linking florid reactive periostitis to Turret exostosis; however, further studies have established Nora’s lesion as an independent pathological entity [3,5]. Some studies have also focused on the genetic characterization of BPOP by using chromosome banding and fluorescence in situ hybridization analyses, and have detected a balanced translocation between chromosomes 1 and 17: t (1;17) (q32;q21) [1]. These differences in opinion regarding the etiology of BPOP prompt questions regarding whether the lesion always originates from an intact cortex, and whether considering only this criterion might lead to misdiagnosis, thus reinforcing the view that a histopathology is a critical tool for diagnosis of Nora’s lesion [5].

Treatment of BPOP depends on the size of the lesion, symptoms, and alterations in vital hand function: if the lesion is small and asymptomatic, no treatment other than follow-up is needed, whereas if the lesion involves pain and functional restriction, surgical treatment is necessary [5]. Although the surgical modality depends on the patient’s requirements and symptoms, and the characteristics of the lesion (size or location), en bloc resection with wide margins (excision of the pseudocapsule over the lesion, periosteal tissue beneath the lesion, and decortication in the underlying periosteum and cortex) decreases the likelihood of recurrence [1,3,7]. In cases in which resection would lead to substantial bone loss or postoperative instability, allografts, plates and screws, or prostheses can be used [1]. Amputation has been proposed in cases in which the mass compromises the surrounding soft tissue and would result in inadequate wound closure and restricted hand functionality [2,5].

In our case, the patient did not indicate any history of trauma at the time when he felt pain or previously, and radiographic examination revealed relevant intraosseous lesions of the medullary canal of the proximal phalanx, with possible pathological fracture of the dorso-radial cortex due to the substantial size of the lesion. The same suspicion was confirmed by MRI findings. Intraoperatively, we performed a wide excision of the lesion, as is performed in case of any bone tumor, and the residual bone gap was covered with grafts from the distal radius. Diagnosis of the BPOP was confirmed on histopathological examination.

The uniqueness of this case is that the lesion was found intramedullary contrary to the other cases, where BPOP is found to arise from the periosteum of the tubular bones and radiographically appears as a broad osseous protuberance of metaphysis, but the underlying bone cortex retains its normal appearance. Hence, suspicion about this diagnosis should be raised even in intramedullary findings.

The recurrence rate is high. Nora et al. (1983) reported a 51% rate of primary recurrence and a 22% rate of secondary recurrence, whereas other studies have reported a recurrence rate of 50%-55% between two months and 10 years [1,2,6]. To decrease postoperative recurrence, one suggested strategy is performing a preoperative bone scan and delaying surgery until the lesion is no longer active [7].

Generally, given the high incidence of recurrence and deficiency of therapy, Nora’s lesion has been suggested to require long-term follow-up after radical surgical excision which gives the patient relief from symptoms. Re-examination of patients every six months for the first two years is recommended; however, patients must be informed of the potential for recurrence over many years [7]. Hence, we suggest that a close patient-doctor relationship should be maintained to diagnose any recurrence on time and treat it accordingly.

## Conclusions

BPOP, or Nora’s lesion, is an unusual, benign, bony pathological entity that poses diagnostic and therapeutic challenges. Although an initial diagnosis of BPOP can be made based on typical radiological signs, only histopathological evidence can precisely confirm the differential diagnosis between BPOP and other benign or malignant bony lesions. Because of the high recurrence rate, surgical excision of the lesion with resection of the underlying periosteum and cortex may decrease the incidence of relapse. Long-term follow-up of patients is necessary, and in cases with repeated recurrence and restriction of hand functionality, amputation may be the definitive treatment.
